# Role of sequence length and functionalization in interactions of bioconjugated peptides with mitomembranes

**DOI:** 10.1116/6.0004197

**Published:** 2025-02-25

**Authors:** Son V. Nguyen, Roy P. Planalp, Harish Vashisth

**Affiliations:** 1 Department of Chemistry, University of New Hampshire, Durham, New Hampshire 03824; 2 Department of Chemical Engineering and Bioengineering, University of New Hampshire, Durham, New Hampshire 03824; 3 Integrated Applied Mathematics Program, University of New Hampshire, Durham, New Hampshire 03824; 4 Molecular and Cellular Biotechnology Program, University of New Hampshire, Durham, New Hampshire 03824

## Abstract

Cell-penetrating peptides are efficient tools for intracellular delivery of a variety of cargoes. In this study, we explored the effect of chain length, side chain chemistry, and the locations of conjugated molecules on the interaction between iron-chelating peptides and a mitochondrial-mimicking membrane. We report that a longer chain length enhanced peptide/membrane interactions, and conjugation at the N-terminus lowered the free-energy barrier for peptide translocation across the membrane. Peptides containing Phe side chains and those containing modified Phe (cyclohexane) side chains showed comparable peptide/membrane energetics and translocation energy barriers. Using steered molecular dynamics (SMD) simulations, we further probed the mechanistic details of translocation of each N-terminated peptide across the membrane and compared their metastable states. At a higher steering velocity, the peptide adopted a compact structure due to frequent π– π interactions among conjugated molecules, but at lower steering velocities, each N-terminated peptide adopted an extended structure. This structure allowed cationic residues to maximize their interactions with phosphate headgroups in the mitomembrane. The hydrophobic residues also formed interactions with the lipid acyl tails, facilitating the passage of peptides across the membrane with decreased free energy barriers. Our results highlight the significance of peptide chain length and conjugation in facilitating peptide transport across the membrane.

## I. INTRODUCTION

Cell-penetrating peptides (CPPs) encompass a diverse group of peptides that can permeate cell membranes without disrupting their integrity or functionality.^1^ They have been studied for their ability to transport therapeutics, macromolecules, and other cargoes into the cellular environment. Several studies have shown that CPPs are efficient tools for intracellular delivery of a variety of cargoes including siRNA,^2,3^ anticancer drugs,^4^ imaging molecules,^5,6^ nanoparticles,^7,8^ and other proteins.^4^ Following the initial identification of the first CPPs (HIV-1 Tat),^9,10^ numerous CPPs have been uncovered or synthesized artificially.^1^ While all CPPs share a common ability to traverse the cell membrane, each of them is unique in length, structural composition, and physical and chemical properties. Thus, CPPs are further classified into categories for their distinctive features. Multiple approaches are used for their classification including origins of the CPPs (protein-derived or synthetic peptides),^11^ physicochemical properties,^12^ cellular toxicity, organelle-targeted, mechanism of entry,^13^ and other features.^11,14,15^ Among various classes of CPPs, mitochondrial-targeting peptides are particularly intriguing due to the complex and important role of the mitochondrion in energy production and multiple cellular functions.^16^

The mitochondrion is a double-membraned organelle with a highly hydrophobic inner membrane and a negative membrane potential ( −180 mV), which limits diffusive transport and permability of molecules commonly used to target cells such as siRNA and low-molecular-weight drugs.^16,17^ Given these challenges, molecules or peptides used to target mitochondria are typically lipophilic and cationic.^18–23^ Small lipophilic cations, such as triphenylphosphonium (TPP), are selectively taken up by the mitomembrane.^24–27^ The uptake mechanism depends on the attraction of positively charged ions, driven by the mitochondrial membrane potential,^21^ which allows them to cross through both the outer and inner mitochondrial membrane.^28,29^ However, in some cases, the energy barrier for hydrophilic compounds to traverse the mitomembrane can be so high that TPP-conjugated molecules cannot overcome.^30^ Thus, an increase in the hydrophobicity of the TPP-conjugated molecules with increasing chain length has been shown to promote cellular uptake.^31^ Mitochondrial-targeting peptides exhibit similar characteristics, being both positively charged and lipophilic.^22,23,32,33^ We have previously studied the spontaneous interaction between an iron-chelating peptide (named SSP peptide) that contains a fluorophore and a mitochondrial-mimicking membrane using atomistic molecular dynamics (MD) and constant-velocity steered MD (cv-SMD) simulations.^34^ Our peptide sequence was based on experimental studies of peptides that demonstrated mitochondrial localization.^22^ In our previous work, we showed that the bioconjugated peptide had a high affinity toward the membrane and the peptide translocation is thermodynamically favorable at a significantly slower steering velocity.

**FIG. 1.**
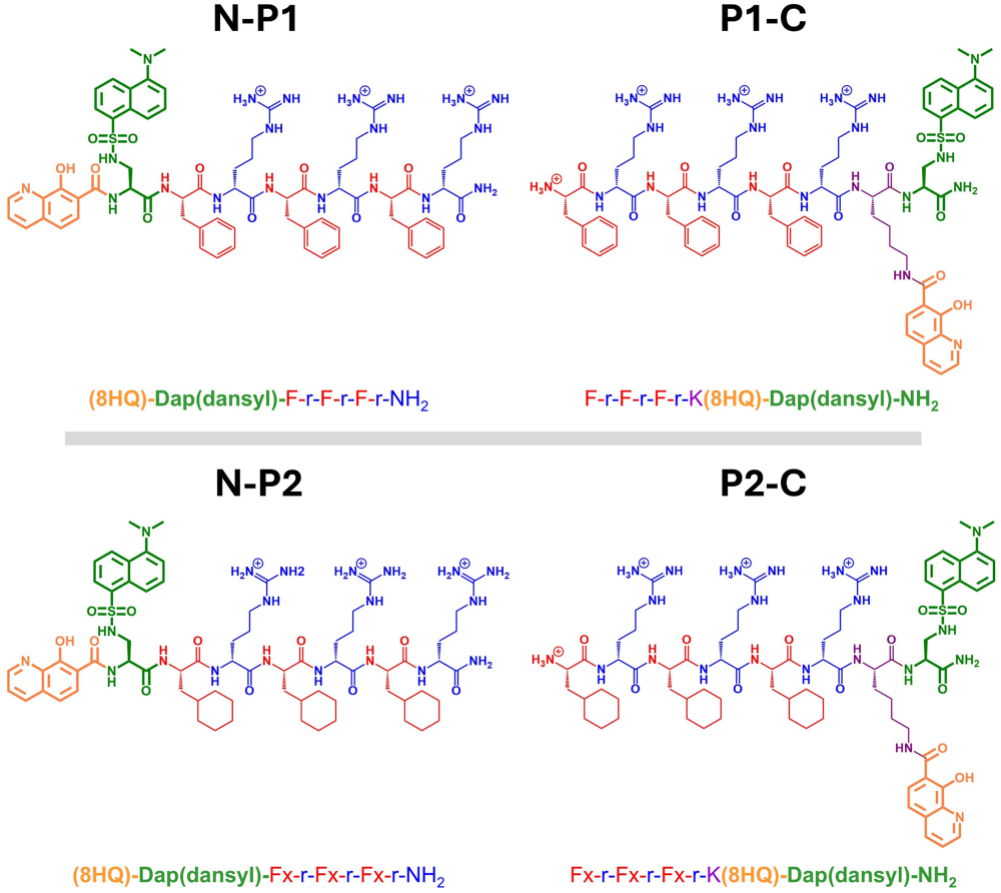
Chemical structures of model peptides. Residues and conjugated molecules are uniquely colored: Phe (F)/modified Phe (Fx), red; (D)-Arg, blue; Lys, purple; 8HQ, orange; Dap(dansyl), green.

In this work, we have designed two peptides (P1 and P2) and first studied their interactions with a mitomembrane using atomistic MD simulations. Both P1 and P2 shared the same sequence [F-r-F-r-F-r, where F is Phe and r is (D)-Arg]. The sequence was based on a class of mitochondrial targeting peptides^33,32^ that shared a residue motif similar to our SSP peptide from the previous work [F-r-K(8HQ)-Dap(dansyl)-NH_2_]^34^ but with longer chain lengths. We used the conventional Phe amino acid (F) in peptide P1 but replaced the benzene ring in the Phe side chain with a cyclohexane (Fx) in peptide P2 as the experimental reports indicated that the peptide with the cyclohexyl-modified Phe residue tends to have higher cellular uptake due to higher lipophilicity.^33,32^ For each peptide, we constructed two versions: One version with the iron chelator and fluorophore conjugated to the N-terminus by the Phe or modified Phe residue (N-P) and another version with the conjugated molecules on the C-terminus by a Lys residue (P-C). The resulting model peptides are as follows: N-P1 [(8HQ)-Dap(dansyl)-F-r-F-r-F-r-NH_2_], P1-C [F-r-F-r-F-r-K(8HQ)-Dap(dansyl)-NH_2_], N-P2 [(8HQ)-Dap(dansyl)-Fx-r-Fx-r-Fx-r-NH_2_], and P2-C [Fx-r-Fx-r-Fx-r-K(8HQ)-Dap(dansyl)-NH_2_] (Fig. 1). These peptides were constructed to test the effect of chain length, side chain chemistry, and the positions of conjugated molecules on peptide/membrane interactions.

Using atomistic MD simulations, we studied the influence of our modifications on the conformational behavior of peptides. We constructed all-atom systems with each system containing a peptide, a mitochondrial-mimicking membrane, adequate aqueous ions, and water molecules. Each peptide was initially oriented so that the plane containing the peptide backbone was relatively parallel to the membrane plane. We subsequently conducted all-atom explicit-solvent conventional MD simulations to investigate spontaneous peptide and membrane interactions. We also employed nonequilibrium constant velocity steered MD (cv-SMD) simulations to study the barrier-mediated translocation process for each peptide. We found that longer peptides had a higher affinity for the membrane compared to shorter peptides. The modification to the Phe side chain had little effect on spontaneous peptide/membrane interaction and during membrane translocation. We identified the preferred penetration mode and the positions of the conjugated molecules for model peptides. The mechanistic insights, revealed by cv-SMD simulations, demonstrates the importance of peptide conformation and metastable states during translocation. The enhancement of hydrophobic interactions between the peptide’s hydrophobic side chains and the lipid acyl tails facilitated peptide transport by reducing the free energy barrier. Overall, our work explored multiple modifications in peptides to further enhance the efficacy of our iron-chelating peptides.

## II. MATERIALS AND METHODS

### A. System setup

We examined the interactions of bioconjugated peptides with a mitochondrial membrane through all-atom MD simulations. We used the graphical user interface (GUI) tool, CHARMM-GUI,^35–38^ to create a mitochondrial membrane containing 280 phospholipids (140 per leaflet). The mitomembrane was comprised of phosphatidylcholine (PC), phosphatidylethanolamine (PE), and cardiolipin (CL)^39^ with a ratio of 10:30:60 for each leaflet. The model peptides (Fig. 1) were created using the Avogadro software^40^ and parameterized using the CHARMM-36m force field.^41^ The conjugated molecules on the peptides were parameterized using the CHARMM General Force Field (CGenFF).^42–45^ To study peptide/membrane interactions, we constructed each system with the peptide aligned along the z-plane of the membrane and translated by 20 Å from the membrane surface. The peptide was initially aligned so that its backbone is relatively parallel to the membrane plane (Fig. S1A in the supplementary material). We solvated each system with the CHARMM modified TIP3P water molecules^41^ based on the original TIP3P water model,^46^ while keeping the membrane domain free of water molecules. We also added counterions (24 sodium ions for P1-C or P2-C systems and 25 sodium ions for N-P1 or N-P2 systems) to neutralize the overall charge of each system.

### B. MD simulations

All MD simulations were conducted following the widely used protocol for membrane-based systems.^47,48,34^ In the first stage, water molecules were equilibrated with constraints placed on all other atoms in the system. Following this stage, the equilibration process targeted the lipid tails while continuing to constrain the other atoms. Subsequently, only the peptide underwent equilibration. For production runs, all constraints were removed. Each system had two replicates, and each trajectory was 0.5 μs long. The temperature and pressure were maintained at 310 K and 1 atm using the Langevin thermostat and the Nosé–Hoover barostat. The pressure coupling was anisotropic with the xy-plane of the membrane kept constant while the z-plane was allowed to fluctuate. The Particle Mesh Eswald (PME) method was used for electrostatic interactions computed with a cutoff of 12.0 Å. All simulations were conducted using the NAMD software^49^ with the CHARMM-36m force field,^50,41^ and simulation trajectories were visualized using the Visual Molecular Dynamics (VMD) software.^51^

### C. Steered MD simulations

The movement of each peptide across the membrane was examined by performing cv-SMD simulations at different steering velocities.^52–54^ During cv-SMD simulations, a steering force was applied on the C ${}_{\alpha }$ atom of the residue to which the conjugated molecules were linked. We created new solvated and ionized systems for each model peptide (Fig. S1B in the supplementary material). In each system, the peptide was positioned outside the membrane within the solvent region, and the length of the simulation domain on the side of the opposite leaflet was increased by 20 Å along the *z*-axis, which is perpendicular to the membrane plane (Fig. S1B in the supplementary material). Each system contained ∼130 000 atoms. All systems underwent energy minimization and equilibration (500 ns) in the NPT ensemble with the peptide atoms harmonically restrained. The steering reaction coordinate (RC) was defined by a vector based on the *z*-coordinate of the pulled C ${}_{\alpha }$ atom in the peptide backbone to the *z*-coordinate of a point located 100 Å along the *z*-axis below the lower leaflet and within the solvent region of the simulation domain.

A harmonic external force was applied on the selected C ${}_{\alpha }$ atom using a spring constant of *k* = 12 kcal/mol ⋅Å ${}^{2}$.^53,55,56,34^ The initial steering velocity was chosen to be 2 Å/ns to cover a distance of 100 Å, resulting in a 50 ns long cv-SMD simulation. We further conducted cv-SMD simulations at significantly lower velocities of 0.5 and 0.1 Å/ns (200 and 1000 ns long simulations, respectively). We applied harmonic constraints on the phosphorus atoms in lipids (force constant k = 1 kcal/mol ⋅Å ${}^{2}$) to prevent membrane movement along the *z*-axis and on the pulled atom (force constant k = 12 kcal/mol ⋅Å ${}^{2}$) to restrict peptide drifting in the xy-plane along the RC. We performed cv-SMD simulations after system equilibration. At velocities of 2 and 0.5 Å/ns, we conducted 10 cv-SMD simulations, but for a significantly slower velocity of 0.1 Å/ns, we conducted three independent cv-SMD simulations. Overall, cv-SMD simulations resulted in a 0.5 μs dataset at the steering velocity of 2 Å/ns, 2 μs dataset at the velocity of 0.5 Å/ns, and 3 μs dataset at the velocity of 0.1 Å/ns.

### D. Potential of mean force calculation

We computed the potential of mean force (PMF) based on the distance along the RC ( r), which increased at a steady rate v, using the relation $\lambda_{t}=\lambda_{0}+vt$ with $\lambda_{0}$ initialized at 0. The external work performed for a nonequilibrium SMD trajectory is described by the following equation:

$$W_{0\to t}=-kv\int_{0}^{t}(r-(\lambda_{0}+vt))dt.$$ (1)

We followed the methodology outlined by Jensen *et al.*^52^ and employed exponential averaging based on Jarzynski’s equality^55–57^ to determine the PMF along the RC based on the work values derived from cv-SMD simulations. The formula for exponential averaging is as follows:

$$△G=-\beta^{-1}ln\bar{exp(-\beta W)},$$ (2)

where $\beta =1/k_{B}T$ with Boltzmann’s constant $k_{B}$ and T temperature. W is the nonequilibrium work performed in each cv-SMD simulation, and △G is the equilibrium-free energy difference.

## III. RESULTS

### A. Peptide and membrane interactions

**FIG. 2.**
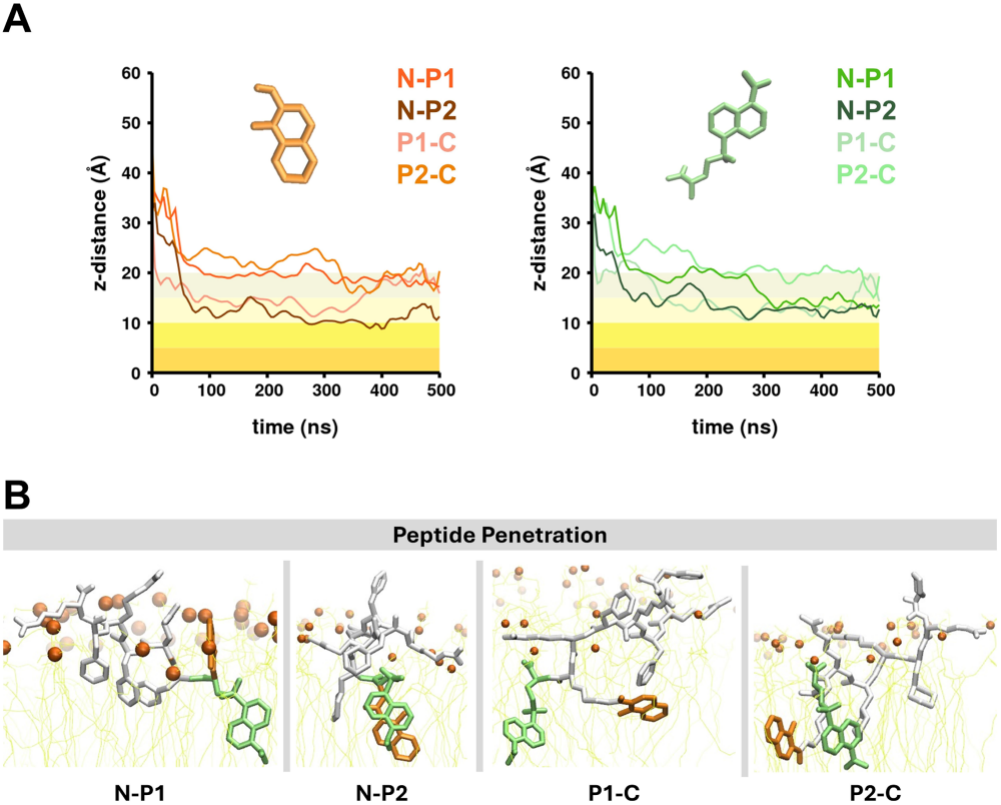
Peptide/membrane interaction analysis from conventional MD simulations. (a) The *z*-distance computed between the center of mass (COM) of the 8HQ (orange, left) and the Dansyl (green, right) relative to the membrane COM for each system. The shaded area represents the membrane, and the color gradient marks the membrane depth every 5 Å. (b) The conformation of each peptide is shown for a typical membrane penetration state.

To study the interaction between each peptide and the membrane, we performed all-atom MD simulations, in which each peptide was placed in the solvent region, outside of the membrane. For each of the four systems, we observed that the peptide spontaneously diffused toward the membrane and initiated penetration into the membrane after forming surface interactions. We also observed for all peptides that the terminus with the conjugated molecules (8HQ and Dansyl) penetrated deeper into the membrane compared to the other terminus [Fig. 2(b)]. A distance analysis between the center of mass of the 8HQ/Dansyl side chain and the center of mass of the membrane was performed for all trajectories. We computed the averaged distances for the 8HQ and Dansyl side chains for each peptide [Fig. 2(a)]. The peptides P1-C and N-P2 exhibited higher penetration depths for both 8HQ and Dansyl side chains (Table S1 in the supplementary material) compared to P2-C and N-P1 peptides. The penetration depth for the Dansyl side chain in N-P1 is higher than the 8HQ side chain, whereas, in P2-C, the penetration depth values for both side chains were similar (Table S1 in the supplementary material). Each peptide exhibited a similar binding state, with one (D)-Arg residue oriented toward the membrane surface as the peptide approached the membrane [Fig. 2(b) and Fig. S2 in the supplementary material]. The averaged RMSD value for each of the four peptides was 7.3 Å ± 0.5 (P1-C), 7.2 Å ± 0.5 (P2-C), 7.2 Å ± 0.5 (N-P1), and 6.6 Å ± 0.8 (N-P2) (Fig. S3A in the supplementary material) compared to the peptide initial conformations. We also computed the buried surface area (BSA) for each peptide. The averaged BSA for each peptide was 906 Å ${}^{2}$ ± 122 (P1-C), 687 Å ${}^{2}$ ± 192 (P2-C), 776 Å ${}^{2}$ ± 174 (N-P1), and 949 Å ${}^{2}$ ± 165 (N-P2) (Fig. S2B in the supplementary material).

We further present the mean nonbonded interaction energy (van der Waals and electrostatic) between all atoms in each peptide and those in the membrane (Fig. S4 in the supplementary material). The mean energy for each system was −824.4 ± 25.1 kcal/mol (P1-C), −714.6 ± 25.0 kcal/mol (P2-C), −799.3 ± 26.8 kcal/mol (N-P1), and −794.6 ± 23.6 kcal/mol (N-P2), verifying that the peptide/membrane interaction was favorable (Table S2 in the supplementary material). The primary contributor to the total nonbonded interaction energy is the electrostatics energy, with (D)-Arg being the major contributor in each system and 8HQ being the least (Table S2 in the supplementary material). The van der Waals interaction energy was the highest for the fluorophore Dansyl (Table S2 in the supplementary material).

### B. Peptide translocation across membrane

To probe the influence of the physical and chemical properties of each peptide on their membrane translocation, we conducted nonequilibrium cv-SMD simulation for each peptide. Based on the findings of conventional MD simulations indicating that the peptide terminus linked with the conjugated molecules (8HQ, Dansyl) exhibited a greater penetration depth, we chose the C ${}_{\alpha }$ atom of the linking residue as the SMD atom for applying the external force to guide the peptide across the membrane. We extracted the force profiles from each cv-SMD trajectory and intergrated the curve to obtain the work required to guide the peptide. Subsequently, we used these work values to compute the potential of mean force (PMF) along the reaction coordinate (RC) employing Jarzynski’s equality [Eq. (2)].

#### 1. Effect of peptide side chain on membrane translocation

**FIG. 3.**
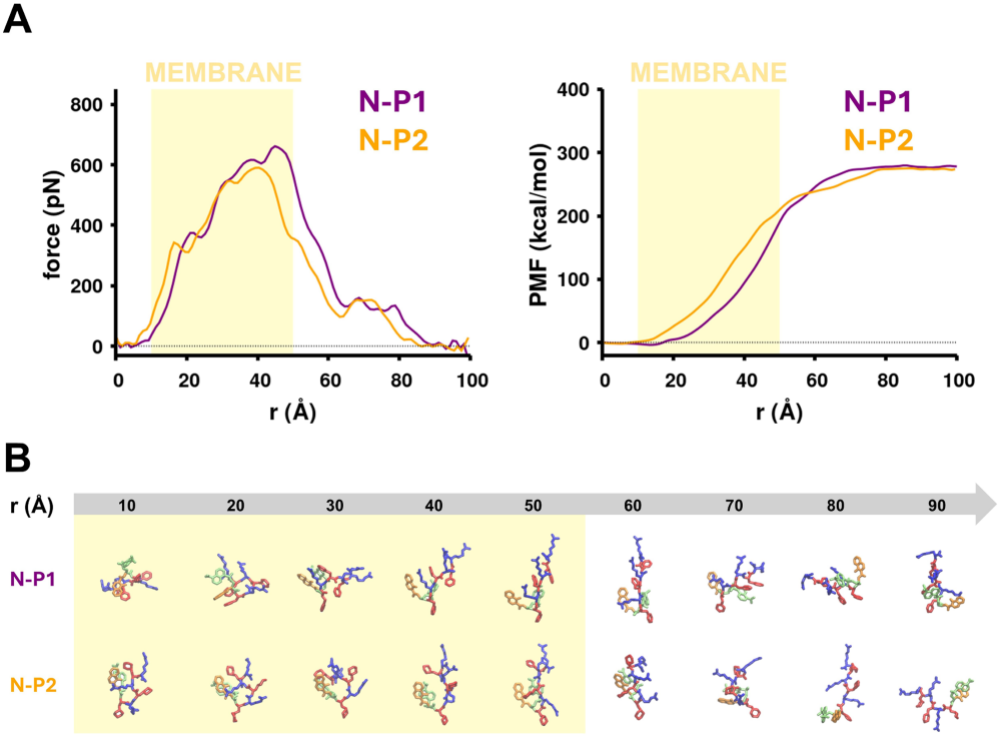
Comparison of the force and free energy profiles and metastable states for translocation of N-P1/N-P2 peptides during cv-SMD simulations at a steering velocity of 2 Å/ns. (a) The averaged (over 10 cv-SMD simulations) translocation force (*left*) and the corresponding free energy profiles (*right*) along the RC for the N-P1 and N-P2 peptides in which the external force is applied to the C ${}_{\alpha }$ atom of the linking residue of the conjugated molecules. The shaded area represents the membrane. (b) Several conformational states along the RC for peptides N-P1 and N-P2. The color scheme is similar to Fig. 1.

For each of the four peptide/membrane systems, we conducted initial cv-SMD simulations with a steering velocity of 2 Å/ns to study the translocation of each peptide across the membrane. Each pair of peptides we compared (N-P1 vs N-P2 and P1-C vs P2-C) shared identical sequence, and the location of conjugated molecules (8HQ, Dansyl), but differed only in the chemistry of the Phe side chain. The peptide P1 represents the conventional Phe amino acid while P2 had a modified side chain with the cyclohexane in place of the benzene ring. At the beginning of each cv-SMD simulation, each peptide was situated outside the membrane in the solvent area, and its interaction with water molecules resulted in a net force of zero [Fig. 3(a) and Fig. S5 in the supplementary material]. As the distance between the peptide and the membrane decreased, the peptide adopted a membrane-binding conformation similar to as observed in conventional MD simulations, where (D)-Arg residues were oriented toward the membrane surface. All force profiles showed an increase in the force values at a distance of 10 Å from the membrane due to long-range electrostatic interactions between the charged residues in the peptide and the phosphate headgroups in lipids. The force values continued to increase until the peptide was situated in the lower leaflet area (at RC = 40–50 Å for each peptide). All force profiles showed a decrease in force values ( <200 pN) due to the disruption of van der Waals interactions between the peptide and the membrane [Fig. 3(a) and Fig. S5 in the supplementary material]. The forces converged to zero beyond 90 Å (Figs. S6 and S7 in the supplementary material), signifying a complete dissociation of the peptide from the membrane.

The values of forces and free energies for peptides N-P1 and N-P2 were comparable at penetration and exit states. The penetration force (force required to dissociate the peptide from the upper leaflet) for N-P1 is ∼660 pN at ∼45 Å and for N-P2 is ∼587 pN at ∼40 Å [Fig. 3(a)]. The exit force (force required to dissociate the peptide from the lower leaflet) for N-P1 is ∼158 pN at ∼70 Å and for N-P2 is ∼154 pN at ∼71 Å [Fig. 3(a)]. The total translocation free energy for both N-P1 and N-P2 are ∼278 ± 51.4 and ∼272 ± 51.4 kcal/mol [Fig. 3(a) and Fig. S8 in the supplementary material]. The peptide pair P1-C and P2-C also shared similar force and free energy values. The penetration force of P1-C is ∼809 pN at ∼43 Å and for P2-C is ∼738 pN at ∼48 Å (Fig. S5 in the supplementary material). The exit force for P1-C is ∼172 pN at ∼78 Å and for P2-C is ∼167 pN at ∼74 Å (Fig. S5 in the supplementary material). The total translocation energy for peptides P1-C and P2-C are ∼307 ± 51.2 and ∼328 ± 51.2 kcal/mol [Fig. 3(a) and Fig. S8 in the supplementary material], respectively.

#### 2. Effect of conjugated molecules on membrane translocation of peptides

The values of forces and free energies for peptide translocation had a noticeable difference among the N-terminated and C-terminated peptides. The penetration force value for P1-C ( ∼809 pN at ∼43 Å) is higher than N-P1 ( ∼660 pN at ∼45 Å), and P2-C ( ∼738 pN at ∼48 Å) is also higher than N-P2 ( ∼587 pN at ∼40 Å) [Fig. 3(a) and Fig. S5 in the supplementary material]. However, the exit forces are similar ( ∼160–170 pN) among all peptides. The translocation free energy barrier of the N-terminated peptide is lower than that of its corresponding C-terminated peptide [Fig. 3(a) and Fig. S5 in the supplementary material]. We also observed that the translocation free energy values for the N-terminated peptides (N-P1 and N-P2) converged during simulations [Fig. 3(a)]. The analysis of peptide conformations along the translocation pathway (RC = 10–50 Å) revealed that the N-terminated peptides (N-P1 and N-P2) exhibited a more compact conformation than the C-terminated peptides (P1-C and P2-C) [Fig. 3(b) and Fig. S5B in the supplementary material]. Among peptides P2-C and N-P2, the 8HQ and Dansyl side chains in N-P2 formed stable π- π interactions, whereas the aromatic side chains in P2-C (modified Phe, 8HQ, Dansyl) showed a lesser degree of interactions with other residues in the peptide until the peptide exit at 50 Å (Fig. S5B in the supplementary material). During translocation, aromatic side chains in N-P1 exhibited multiple π– π interactions (between Phe and 8HQ at 10–20 Å, and between 8HQ and Dansyl at 30 Å) [Fig. 3(b)]. The peptide P1-C had a more linear conformation during translocation with a π– π interaction between 8HQ and Dansyl (Fig. S5B in the supplementary material).

#### 3. Mechanism of membrane translocation of N-terminated peptides

**FIG. 4.**
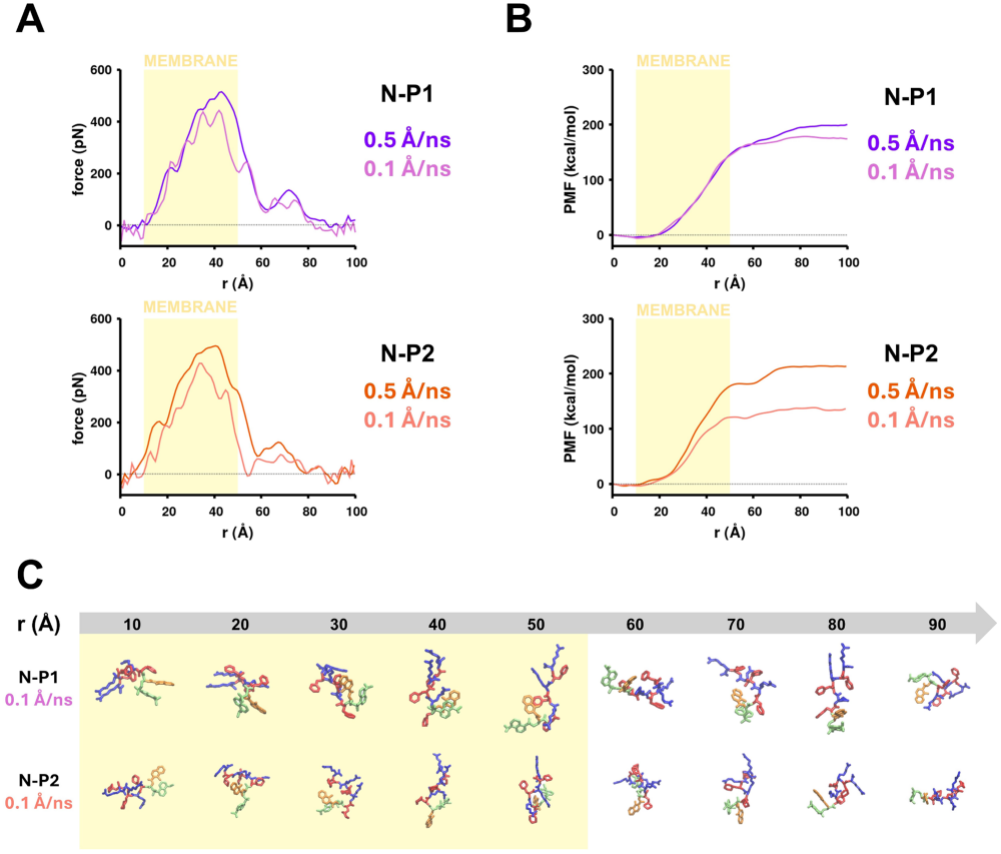
Comparison of the force and free energy profiles and metastable states of N-terminated peptide translocation during cv-SMD simulations at steering velocities of 0.5 and 0.1 Å/ns. (a) The averaged translocation force along the RC for peptides N-P1 (*top*) and N-P2 (*bottom*) in which the external force is applied to the C ${}_{\alpha }$ atom of the linking residue of the conjugated molecules. (b) The corresponding free energy profiles for peptides N-P1 (*top*) and N-P2 (*bottom*). The shaded area represents the membrane. (c) Conformational states along the RC for peptides N-P1 and N-P2 at a steering velocity of 0.1 Å/ns. The color scheme is similar to Fig. 1.

Once we determined that the N-terminated peptides (N-P1 and N-P2) exhibited lower translocation free energy barriers across the membrane, we further analyzed the translocation mechanism of these peptides in cv-SMD simulations at steering velocities of 2, 0.5, and 0.1 Å/ns. The peptides were initially located in the solvent and pulled along the RC ( z-axis vector perpendicular to the membrane plane) across the membrane. In all trajectories at various steering velocities, the binding conformation, where (D)-Arg interacted with the membrane surface, is consistently observed in both peptides N-P1 and N-P2 [Figs. 3(b), 4B, and S9 in the supplementary material]. At a steering velocity of 0.5 Å/ns, the penetration force for N-P1 dropped to ∼517 pN at ∼45 Å, and the exit force dropped to ∼134 pN at ∼70 Å [Fig. 4(a)]. The penetration force for N-P2 dropped to ∼496 pN at ∼40 Å, and the exit force dropped to ∼121 pN at ∼71 Å [Fig. 4(a)]. All force profiles converged at RC = 90 Å (Figs. S10 and S11 in the supplementary material). At a steering velocity of 0.1 Å/ns, the translocation free energy barrier was reduced by ∼22 kcal/mol (compared to 0.5 Å/ns) for the peptide N-P1 and ∼76 kcal/mol (compared to 0.5 Å/ns) for the peptide N-P2 [Figs. 4(a), S10 and S11 in the supplementary material]. At a velocity of 2 Å/ns, each peptide N-P1 and N-P2 had a more compact conformation along the translocation pathway [Fig. 3(b)]. However, at a velocity of 0.1 Å/ns, each peptide had an extended structure [Fig. 4(b)], with the (D)-Arg on the C-terminus pointed toward the leaflet in which the peptide entered and the N-terminus facing the opposite leaflet. Since the N-terminus is more hydrophobic (Phe/Modified Phe, 8HQ, Dansyl) and the C-terminus is more cationic, this linear structure allowed the cationic residues to prolong their interactions with the penetrated leaflet while the hydrophobic residues interacted with the hydrophobic lipid acyl tails.

## IV. DISCUSSION

We used all-atom unbiased MD simulations to study spontaneous peptide-membrane interactions and cv-SMD simulations to study the translocation mechanisms of model peptides differing in their side chains or the locations of conjugated molecules (8HQ, Dansyl). The peptide structures were chosen from a class of mitochondrial-targeting peptides,^33,32^ in which the peptide sequences and residues are similar to, but four amino acids longer than, the SSP peptide in our previous study.^34^ Thus, we developed models of these peptides to study the effect of chain length, side chain chemistry, and locations of conjugated molecules on their interactions with the mitomembrane.

We found that increasing the length of each peptide improved its interaction energy with the membrane but also increased its translocation free energy barrier. More cationic residues [from protonated Phe residues and (D)-Arg] tripled the electrostatic energy that drives peptide localization to the membrane (Fig. S4 in the supplementary material). The van der Waals interaction that contributed to penetration of each peptide also tripled due to the presence of more aromatic residues (Phe/Modified Phe) (Fig. S4 in the supplementary material). A longer peptide length also allowed the bulkier aromatic side chains (8HQ and Dansyl) to have a higher penetration depth into the membrane hydrophobic core (Table S1 in the supplementary material) compared to the SSP peptide.^34^ The membrane-binding states of all peptides in this study are similar to the SSP peptide,^34^ with the cationic side chains pointed toward the membrane surface (Fig. S2 in the supplementary material). The peptide conformations during the penetration state, however, are more similar among longer peptides. All conventional MD trajectories showed that the terminus that was linked to the conjugated molecules (8HQ and Dansyl) was more likely to penetrate the membrane and remain within the hydrophobic core than the other terminus [Fig. 2(b)]. Based on the findings from MD simulations, we hypothesized that should translocation occur, the peptide would cross to the other leaflet from the terminus linked to the conjugated molecules. We created four systems for cv-SMD simulations and applied the external force on the C ${}_{\alpha }$ atom of the residue linked to the conjugated molecules and pulled each peptide across the membrane. The translocation free energy barriers for longer peptides are higher than their shorter counterpart SSP peptide, which is reasonable given that the longer peptides had higher electrostatic energy that should be overcome for penetration to occur.

Peptides with Phe side chains and those with modified Phe (cyclohexane) sidechains showed similar energetics related to peptide/membrane interactions and translocation energy barriers. The contribution to the total nonbonded interaction energy by Phe or the modified Phe residue in four systems is ∼−120.5 kcal/mol (Phe, P1-C), ∼−155.0 kcal/mol (modified Phe, P2-C), ∼−105.0 kcal/mol (Phe, N-P1), and ∼−102.6 kcal/mol (modified Phe, N-P2). The energy values did not vary significantly within the C-terminated pairs (P1-C, P2-C) and the N-terminated pairs (N-P1, N-P2). Some differences in transport properties of peptides observed in our simulations and in previous experimental work^33^ could be due to the complexity of cellular membranes in different cell lines in comparison to simpler mitochondrial-mimicking membrane models employed in simulation studies. In experimental studies, each cell line showed different cellular uptake profiles for peptides containing the Phe side chains and peptides containing the modified (cyclohexane) side chains. While all cell lines showed higher uptake for peptides with the modified Phe side chains compared to those with Phe side chains, in cell line MCF-7, the deviation between cellular uptakes for the two peptides is less significant compared to other cell lines. Moreover, benzene and cyclohexane are similar in that they both contain six carbon atoms arranged in cyclical structures although benzene differs in its aromaticity, resulting in a more constrained structure. While benzene and cyclohexane are both hydrophobic, benzene has been shown to have a more favorable interaction with water molecules.^58^ Thus, side chains containing cyclohexane are more lipophilic and are expected to improve cellular uptake. While cellular uptake studies of these peptides showed that peptides containing modified Phe side chains (cyclohexane) had a higher rate of cellular uptake than peptides containing Phe side chains, cellular uptake of peptides varied among different cell lines, and the membrane localization is mostly dominated by a charge-driven mechanism of uptake instead of lipophilicity.^33,32^ The total nonbonded interaction energy calculated from MD simulations also showed that the Phe and modified Phe side chains in the C-terminated peptides had higher interaction energies with the membrane than the N-terminated peptides. This is because a Phe/modified Phe side chain in the N-terminated peptides being adjacent to the conjugated molecules is more likely to interact with the conjugated molecules than the membrane. In terms of membrane translocation, the penetration and exit force values, as well as the total translocation free energy barriers at a velocity of 2 Å/ns, are comparable for the C-terminated pair and the N-terminated pair [Figs. 3(a) and 3(b)]. Even at lower steering velocities, the force and free energy values did not deviate significantly among the N-terminated pair.

On the other hand, conjugation of the chelator (8HQ) and fluorophore (Dansyl) at the N-terminus resulted in a lower translocation free energy than their conjugation at the C-terminus. By conjugating the chelator and the fluorophore at the N-terminus, we eliminated the need to include the Lys residue to preserve the sequence motif for the model peptide. This reduced the molecular weight and allowed the peptide to be more compact during membrane translocation. During cv-SMD simulations at 2 Å/ns, the C-terminated peptides were observed to be less compact than their N-terminated counterparts. The longer chain of Lys allowed for a greater degree of freedom, leading to more membrane disruption as the peptide moved across the membrane. The smaller N-terminated peptides further optimized interactions within the aromatic groups of the peptide, causing a lower disruption of the membrane.

We further tested the effect of lower steering velocities on the translocation of N-terminated peptides that showed lower translocation free energies. By reducing the velocity from 2 to 0.1 Å/ns, the free energy barriers decreased by ∼100 kcal/mol for both N-P1 and N-P2 [Fig. 4(b)]. Mechanistic analysis revealed that as the peptides (N-P1 and N-P2) crossed the membrane, they adopted a more extended conformation [Fig. 4(c)]. The terminus with the conjugated molecules slowly moved toward the other leaflet while the cationic side chains along the N-terminated peptides continued to interact with the phosphate groups in the leaflet that the peptide penetrated. This conformation of the N-terminated peptides minimized the disruption of the membrane topology. When the N-terminated peptides moved across the membrane at a higher velocity, a more compact structure of the peptides would lower the interaction surface area, limiting abrupt disruption of the membrane. As the steering velocity decreases, the N-terminated peptides enhance hydrophobic interactions with the membrane core. This adjustment allows the cationic residues to reconfigure spontaneously, facilitating the movement of peptides across the membrane.

Our work has highlighted the importance of structure in the design of cell-penetrating peptides. The methodology used in this work (conventional MD simulations and SMD simulations) can be applied for studying molecular transport of other peptides across the membrane, but we note that the peptide transport is studied along a one-dimensional reaction coordinate by applying the SMD simulation method, and therefore, the energy barrier and the mechanism for peptide translocation are also dependent upon the selection of the reaction coordinate. We chose the reaction coordinate based on the residue groups that had a more favorable interaction with the hydrophobic region of the membrane. However, including all degrees of freedom to be sampled will likely lead to a multidimensional reaction coordinate and may require usage of other enhanced sampling methods (e.g., metadynamics, umbrella sampling, etc.) for obtaining an improved estimation of free energy barriers and transport mechanisms.

## V. CONCLUSION

Building on our previous work,^34^ this study explored the effect of chain length, side chain chemistry, and location of conjugated molecules on peptide interaction and translocation across a mitochondrial-mimicking membrane. We constructed four iron-chelating peptides containing a fluorophore: (8HQ)-Dap(dansyl)-F-r-F-r-F-r-NH_2_ (N-P1), (8HQ)-Dap(dansyl)-Fx-r-Fx-r-Fx-r-NH_2_ (N-P2), F-r-F-r-F-r-K(8HQ)-Dap(dansyl)-NH_2_ (P1-C), and Fx-r-Fx-r-Fx-r-K(8HQ)-Dap(dansyl)-NH_2_ (P2-C). We increased the chain length, modified the benzene ring in Phe to cyclohexane, and changed the site where the chelator and fluorophore are conjugated on the peptide. We studied spontaneous interactions of peptides with the membrane as well as the membrane translocation mechanism of each peptide. We reported preferred penetration states, the evolution of the chelator/fluorophore with respect to the membrane, and nonbonded interactions between the peptide and the membrane in conventional MD simulations. We found that the peptide/membrane interaction is highly favorable with a longer peptide length and that the terminus linked to the conjugated molecules had more affinity toward the membrane core. We hypothesized that the peptides were more likely to translocate the membrane from the aforementioned terminus and further performed nonequilibrium cv-SMD simulations at various steering velocities to compare their translocation free energy values. We showed that while altering the side chain of Phe did not significantly influence the peptide interaction and translocation mechanisms, conjugation of the chelator and the fluorophore at the N-terminus resulted in lower translocation free energy barriers. By lowering the steering velocity during translocation, we observed that the peptide adopted a more extended structure, allowing the aromatic side chains to interact with the lipid acyl tails and spontaneously reconfigurate the cationic residues, preventing disruption of the highly favorable interactions between the peptide’s cationic residues and membrane phosphate headgroups.

## SUPPLEMENTARY MATERIAL

See the supplementary material for the system setup, conformational states of peptides during MD simulations, RMSD calculations, BSA calculations, the nonbonded interaction energy calculations, force profiles, force convergence data from cv-SMD simulations, and free energy profiles with error bars from cv-SMD simulations.

## Data Availability

The data that support the findings of this study are available within the article and its supplementary material.
